# Visual Detection of Denatured Glutathione Peptides: A Facile Method to Visibly Detect Heat Stressed Biomolecules

**DOI:** 10.1038/s41598-017-02899-3

**Published:** 2017-06-01

**Authors:** Monique J. Farrell, Robert J. Reaume, Aswini K. Pradhan

**Affiliations:** Center for Materials Research, Norfolk State University, 700 Park Ave., Norfolk, VA 23504 USA

## Abstract

Every year pharmaceutical companies use significant resources to mitigate aggregation of pharmaceutical drug products. Specifically, peptides and proteins that have been denatured or degraded can lead to adverse patient reactions such as undesired immune responses. Current methods to detect aggregation of biological molecules are limited to costly and time consuming processes such as high pressure liquid chromatography, ultrahigh pressure liquid chromatography and SDS-PAGE gels. Aggregation of pharmaceutical drug products can occur during manufacturing, processing, packaging, shipment and storage. Therefore, a facile in solution detection method was evaluated to visually detect denatured glutathione peptides, utilizing gold nanoparticle aggregation via 3-Aminopropyltreithoxysilane. Glutathione was denatured using a 70 °C water bath to create an accelerated heat stressed environment. The peptide, gold nanoparticle and aminosilane solution was then characterized via, UV-Vis spectroscopy, FTIR spectroscopy, dynamic light scattering and scanning electron microscopy. Captured images and resulting absorbance spectra of the gold nanoparticle, glutathione, and aminosilane complex demonstrated visual color changes detectable with the human eye as a function of the denaturation time. This work serves as an extended proof of concept for fast in solution detection methods for glutathione peptides that have experienced heat stress.

## Introduction

Proteins and peptides are key components of biological functions and are essential to the biochemical reactions necessary for life^1–3^. The building blocks for these macromolecules are comprised of 20 amino acids linked together in various combinations through poly-peptide bonds. These structures then fold and combine to form tertiary and quaternary structures, ultimately enabling specific biological functions^4–6^. Peptides in particular have been found to have antimicrobial properties and provide assistance in biological functions such as immune response regulation and host defense^7^. In addition to immunity, they also perform functions facilitated mainly through cell signaling and transduction such as blood pressure and blood volume regulation via the natriuretic peptide family^8, 9^.

As previously stated, proteins and peptides are essential for biological functions. However, if the biological macromolecules have been denatured or experienced significant stress, the intended medicinal effects can become compromised. Specifically, the occurrence of peptide and protein aggregation in biological systems can cause diseases and in pharmaceutical drug products induce adverse immunological responses^10–12^. For example, several studies have linked the occurrence of excess protein and aggregated protein deposition with diseases. One such formation that has been extensively studied is known as amyloid-fibril aggregation, which causes a disease called amyloidosis^13, 14^. This protein deposition disease is characterized by the formation of fibrillar aggregate deposits^15^. These fibrillar aggregates are comprised of a peptide or protein, specific to a certain disease^16, 17^. Early studies performed in the 1960’s injected monomer and higher order molecular weight protein particulates of bovine gamma globulin into rodents. The studies concluded that mitigating the higher order aggregates and particulates, decreased immune responses in the rodents tested^18, 19^. During this time other work also evaluated the effects of aggregated human gamma globulin, formed during commercial preparations and product variations as a function of manufacture source. The aggregates within the trials were found to trigger *in vivo* histamine release and the formation of isoantibodies^20^. Although an important case study, it is critical to note that the human gamma globulin was isolated from the plasma of thousands of healthy volunteers resulting in a heterogeneous product. The non-homogenous nature of the products with respect to percent total gamma globulin varied from 94–99%, is another factor outside of aggregation that may have induced the observed immune responses in patients. Modern day therapeutics that are recombinant DNA based have minimized the issue of heterogeneity, however aggregates are still present to some extent in all drug formulations and need to be studied further. While the presence of aggregated biological macromolecules can occur *in vivo*, this work focuses on the detection of *in vitro* peptide aggregation.

Within pharmaceutical drug products, aggregates of proteins and peptides can form during processing, manufacturing and transport. During this time the protein or peptide may undergo conditions which impact the chemical and physical properties of interest, affecting long term drug stability and product lifetime^21, 22^. Specifically, several factors which can cause aggregation include but are not limited to shaking, shearing, temperature, pH level, and protein concentration^23, 24^. The denaturation of proteins and peptides can be categorized into reversible and irreversible aggregation. Reversible aggregation is a state in which the proteins and or peptides have aggregated, but over time dissociation and reversion back to the native and monomeric forms occur^25–27^. Irreversible aggregates do not revert back to their monomeric forms and can be caused by the rearrangement of disulfide bonds or the formation of new structures due to hydrophobic association; all of which can have a direct impact on immunogenicity^28, 29^. For the purpose of this work only irreversible aggregates induced via an accelerated heat stress will be considered. This paper does not evaluate the impact of aggregate levels on immunogenicity, but seeks to create a useful technique to assess aggregate levels.

With the commercializing of the human growth hormone (hGH), a clinical study, which correlated the level of aggregates within formulations and the immune responses of patients were explored. These studies compared the patients who were given earlier preparations of high level aggregate hGH products (>20%), to patients using formulations taken from the improved method containing less than 10% aggregates. Due to aggregates being present in both high and low level formulations, antibody responses were observed in both but took either a persistent or transient form^30^. It is important to note that the hGH aggregates formed and administered to patients were related to inefficiencies during production purification processes, as opposed to external stresses like heating or shaking. Specifically, the hGH protein in this study was isolated from cadavers (not made recombinantly) and so the observed impact on immunogenicity may differ from the modern products available today. However, this case study does serve as a clear example of manufacturing and processing considerations of pharmaceutical drug products and their impact on patient care. Clinical studies completed by companies who manufacture recombinant interferon beta (IFNbβ), for treatment of multiple sclerosis also observed immunogenicity in their patients^31^. Third party researchers utilized the results from these patient observations, to discuss factors such as product quality and aggregate levels. Within this work, the lowest of the reported cases of immunogenicity was linked to the company with the smallest percentage of aggregates and particulate matter in their drug formulation^32, 33^. The previously described IFNbβ study recently highlights a potential relationship between levels of aggregates and reported cases of immunogenicity. However very little information is currently available that clearly links the immunogenic risk of aggregates found in modern day therapeutics using recombinant processes and manufacturing techniques. Although extended case studies are still needed to evaluate the adverse effects of aggregated or denatured biomolecules on patients, aggregate formation is still an issue. Therefore a method to visually and quickly detect the analytes in a denatured state is crucial.

This work is motivated by several adverse effects that may occur in patients who have been exposed to denatured or non-native biological structures. Specifically, peptides and proteins that have been denatured or degraded can lead to adverse patient reactions such as undesired immune responses^34–36^. In addition, other biological responses that decrease the potency of the drug product can occur^37^. This work is especially impactful as exploration of peptides for applications in diagnostics and therapies has increased. This is due to high costs associated with bringing new small molecule and protein drugs to market^38^. Therefore, with an expected increase in the amount of pharmaceutical drugs and therapies that are peptide based, it is necessary to evaluate simple and cost effective methods to visually characterize peptides. This work explores the visual detection of glutathione (GSH) peptides that have been denatured via an accelerated heat stressed environment, using the artificially induced aggregation of gold nanoparticles (AuNps) via 3-Aminopropyltreithoxysilane (APTES). This will serve as a continued proof of concept to visually detect biological analytes.

## Results

**Figure 1:**
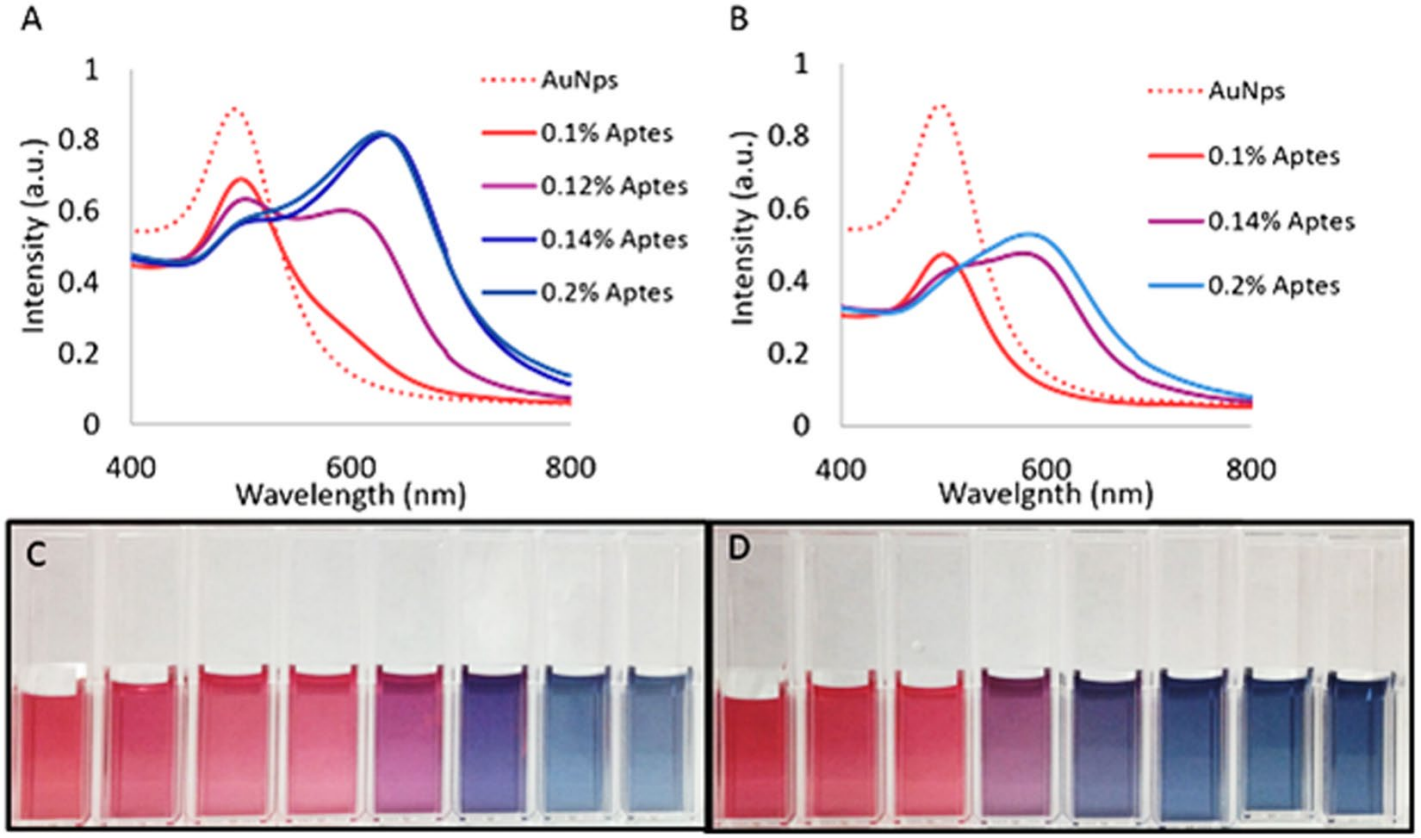
Displays the UV-vis absorbance spectra for the gold nanoparticle and glutathione solutions in the presence of 0.1–0.2% APTES. (**A**) 3.07 mg/mL GSH. (**B**) 30.7 pg/mL GSH. Resulting trials hues from left to right: AuNps, AuNps/GSH, 0.1%, 0.12%, 0.14%, 0.16%, 0.18%, 0.2% (APTES). (**C**) 3.07 mg/mL GSH trial and (**D**) 30.7 pg/mL GSH trial.

Glutathione is a tripeptide essential to life and is produced in the body. It contributes/regulates antioxidant defense, signal transduction, some metabolic functions, immune responses and several other biological functions^39^. The role of antioxidant defense is critical as oxidative stress has been linked to several diseases such as certain cancers, multiple sclerosis, premature aging and other illnesses^40^. Due to glutathione’s small size and prevalence in nature, it serves as a model peptide for evaluation of this detection method for smaller biological analytes. Figure 1 displays the ultraviolet-visible absorbance spectra and images of the AuNps, GSH and APTES solutions. The trials were completed by first mixing 0.1 mL of a no heat stress GSH sample, with 0.8 mL of the AuNp colloidal solution. Subsequently, 0.1 mL of APTES solutions ranging from 0.1–0.2% by volume was added to the solution. For the purpose of this study, the APTES and GSH concentrations were achieved via serial dilution in deionized water. In Fig. 1A we see a systematic red shift in the absorbance spectra of the 3.07 mg/mL GSH trials, as a function of the APTES concentration. The resulting hues of the 3.07 mg/mL GSH trials containing 0.1% and 0.12% correspond to a red hue. However, as the concentration of APTES is increased, a magenta and purple hue is observed at 0.14% and 0.16% APTES. Similarly, a red shift was observed in the 0.18% and 0.2% APTES concentrations in which the resulting solution hues exhibited a blue color. A similar trend is observed within the 30.7 pg/mL GSH trials displayed in Fig. 1B, in which the same procedure was employed. In Fig. 1C and D, we observe systemic red shifts in the absorbance spectra of the solutions as the concentration of APTES is increased, corresponding to a clear progression of hues from red to purple to blue.

**Figure 2:**
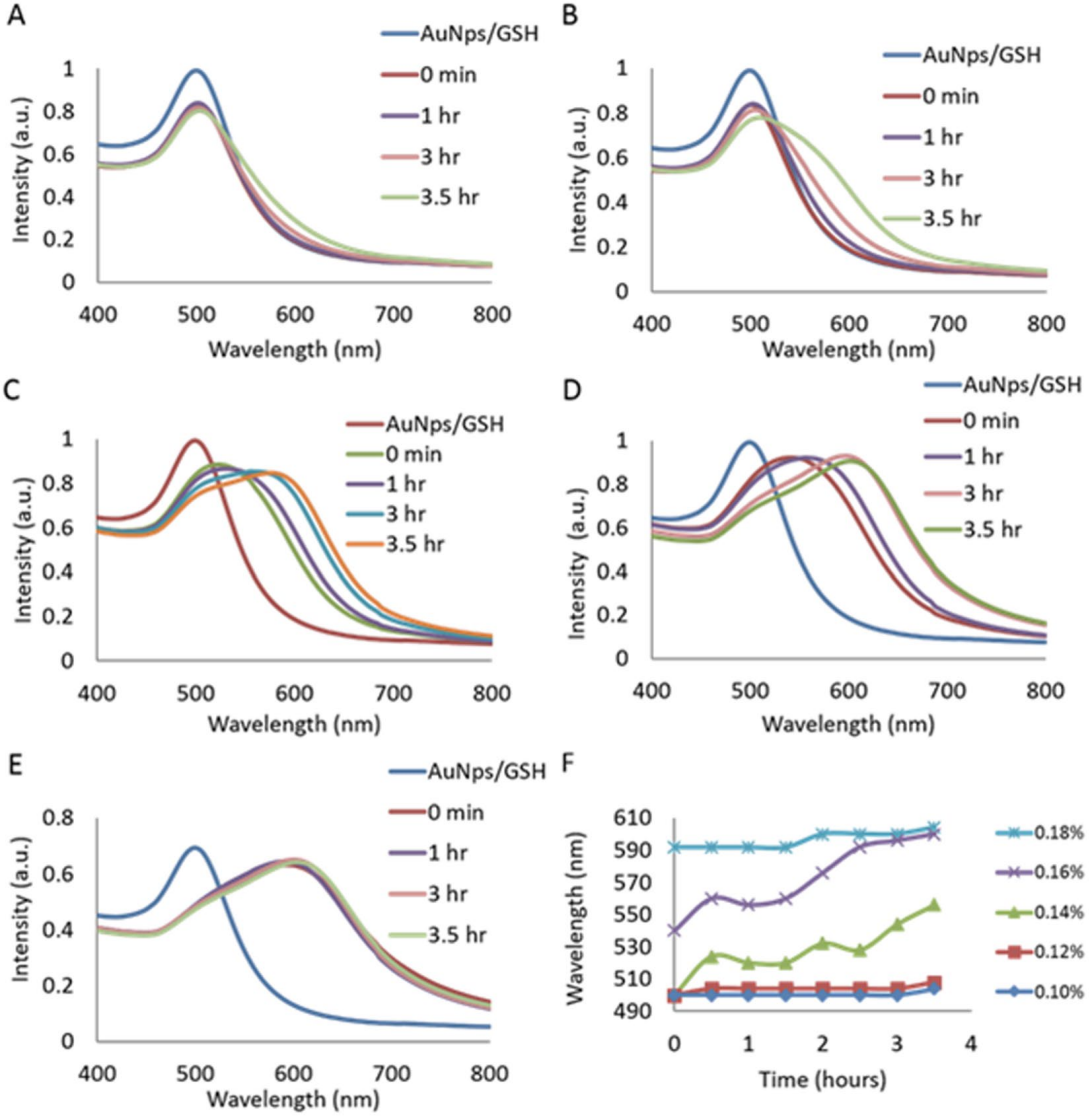
Absorbance spectra of the AuNps, GSH, and APTES solutions over a 3.5 hour GSH peptide denaturation, via a 70 °C accelerated heat stress study. (**A**) 0.1% APTES. (**B**) 0.12% APTES. (**C**) 0.14% APTES. (**D)** 0.16% APTES. (**E**) 0.18% APTES. (**F**) Shows the change in the max peak wavelength as a function of denaturation time for each concentration of APTES.

In Fig. 1 we established sensitivity of the native GSH and gold nanoparticle complex to the presence of APTES. The resulting solutions confirmed our ability to induce the full spectrum of hues achievable using gold nanoparticles, by modulating the concentration of APTES in solution in the presence of GSH. To evaluate the potential for visual detection of denatured glutathione, an accelerated heat stress study was executed and characterized. As described within the experimental section, the GSH was denatured utilizing a heat bath at 70 °C, mixed with the gold nanoparticle solution and 100 uL of each respective APTES concentration. Figure 2 displays the resulting absorbance spectra of the AuNp, GSH, and APTES solutions over a 3.5 hour accelerated heat stress exposure time. At the lowest tested concentration of 0.1% APTES, during the first 3 hours the max peak wavelength of the control and experimental trials were maintained at approximately 500 nm. At 3.5 hours of accelerated heat stress, the absorbance spectra in Fig. 2A did show a distinct redshift, via a peak broadening. Increasing the concentration of APTES from 0.1% to 0.12%, the changes in the max peak wavelength increased throughout the accelerated heat stress study (Fig. 2B).

In Fig. 2B we observe a systematic increase in the max peak wavelength and corresponding peak broadening at 1 hour of heat stress for the 0.12% APTES trial. This continuous red shift is sustained throughout the remainder of the time trial. The extension of the max peaks wavelengths within the displayed absorbance spectra towards the near infrared region is exaggerated as the APTES concentration is increased from 0.1 to 0.16% APTES. In Fig. 2C and D not only do we observe extreme peak broadening, but in both trials the max absorbance peak wavelength also shifts greater than 80 nm. The highest concentration of 0.18% APTES demonstrated a trend similar to the 0.1% APTES trials, in which the max peak wavelength was constant for the majority of the accelerated heat stress study. The initial color of the AuNps, GSH and 0.18% APTES solution is blue and so as the heat stress is continued there is no color transition (Fig. 2E). For the concentrations tested, the optimal APTES concentrations are therefore between 0.12% and 0.16%. In Fig. 2F, the changes in the absorbance max peak wavelength as a function of the denaturation times are displayed. This figure highlights the effects of APTES concentration on the absorbance spectra of the trial samples. In Fig. 2F we observed the steepest slopes within the 0.14% and 0.16% APTES concentrations. This is consistent with the greatest amount of change observed with respect to the max peak wavelength. This information is critical to designing and choosing parameters for future in solution detection methods; specific to drug product concentration and allowable levels of denaturation. For example, if a particular drug can withstand extended amounts of heat stress then a smaller concentration of APTES such as 0.1% should be utilized. This would be an ideal concentration to start with, as a significant change within the absorbance spectra was not observed until 3.5 hours of heat stress. Alternately if the drug product is more sensitive to heat stress, then a higher concentration of APTES such as 0.16% should be employed as a significant change in the max peak wavelength took a shorter time of 2.5 hours.

**Figure 3:**
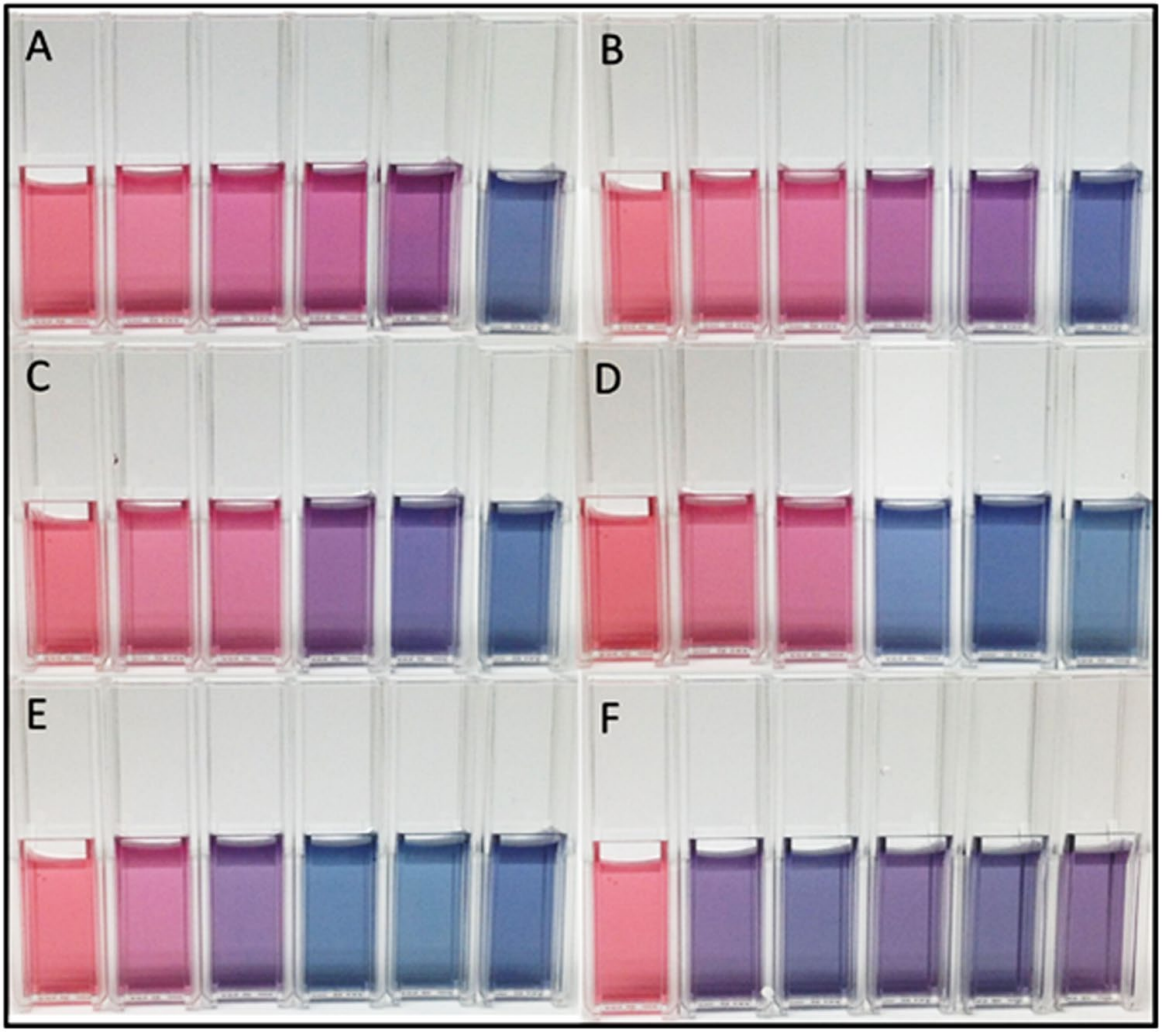
Captured images of the AuNps/GSH/APTES solutions over a 3.5 hour GSH peptide denaturaation via 70 °C accelerated heat stress. From left to right: AuNps, 0.1% APTES, 0.12% APTES, 0.14% APTES, 0.16% APTES, 0.18% APTES. (**A**) 0 minute, (**B**) 1 hour, (**C**) 2.5 hours, (**D**) 3 hours, (**E**) 3.5 hours, and (**F**) 4 hours no APTES.

The changes in absorbance spectra displayed in Fig. 3 correlate to visual color changes in the solutions as a function of the concentration of APTES and the extent of glutathione denaturation. Figure 3A shows the solution colors for the control group containing 100 uL of the native GSH, AuNps and respective APTES concentrations ranging from 0.1–0.18%. After 1 hour of heat stress, the trial containing 0.14% APTES transitioned from a red hue to a purple hue, thus capturing visually the occurrence of GSH denaturation (Fig. 3B). At 2.5 hours of the accelerated heat stress (Fig. 3C), the trial containing 0.16% APTES displays a blue hue. This is in stark contrast to the original purple hue observed at the 0 hour and 1 hour trials within Fig. 3A and B. In Fig. 3C, the solution color resulting in GSH aliquots taken after 2.5 hours of heat stress for the 0.16% APTES concentration begins to turn a blue/purple hue. However, after 30 more minutes (Fig. 3D), we observe a very distinguished blue hue at the 0.14% and 0.16% APTES concentrations. At 3.5 hours (Fig. 3E) we see a visual detection and color transition within the 0.12% APTES concentration, from red to a distinctive purple hue. Figure 3 clearly shows the visual dependence of the AuNps, GSH, and APTES solution on the exposure of the glutathione to a heat stressed environment. It also supports the feasibility of our system to visually detect GSH that has been denatured.

### Proposed Mechanism

**Figure 4:**
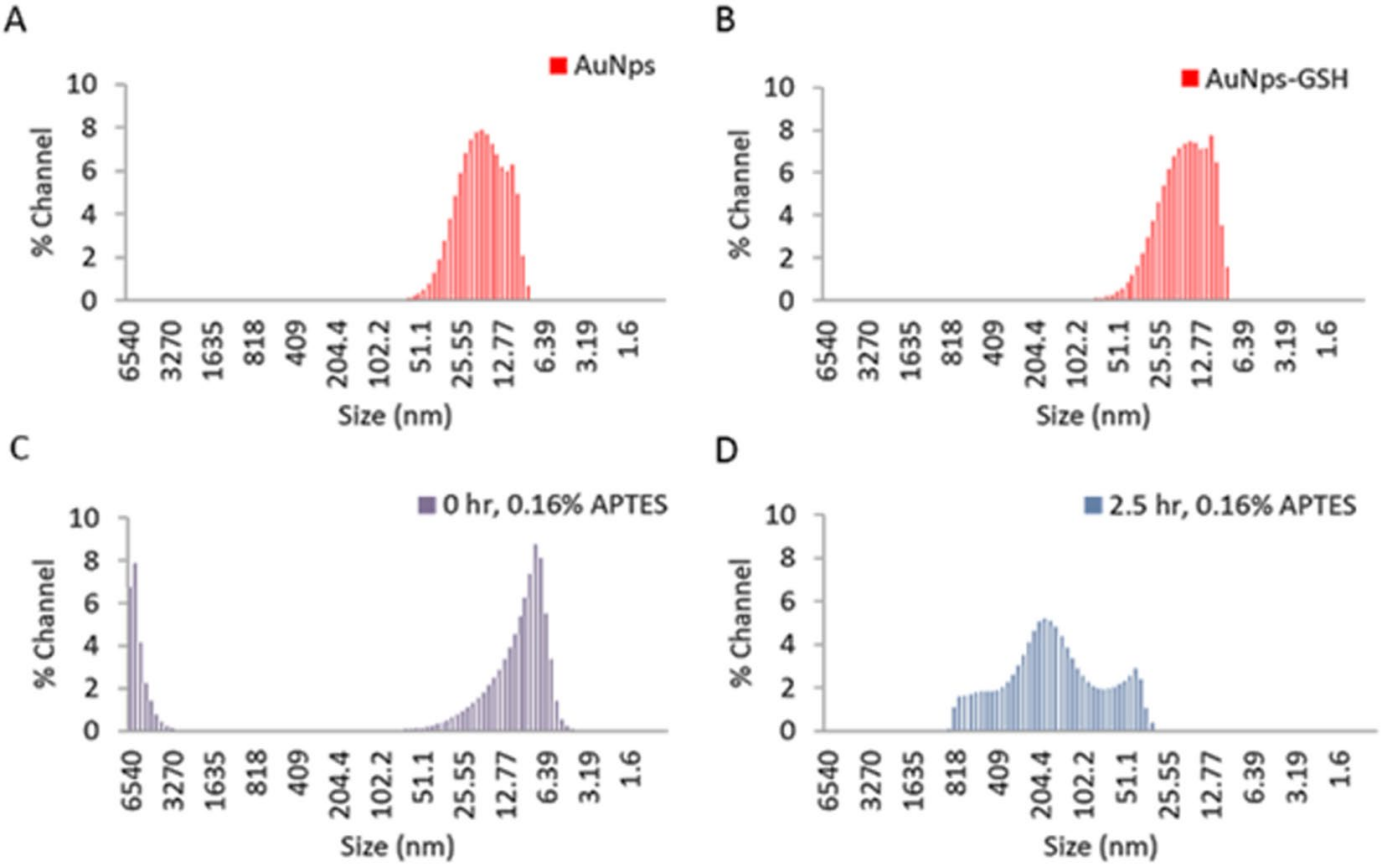
Size and size distribution plots of the AuNps/GSH/APTES solutions as determined by dynamic light scattering measurements. (**A**) Control consisting of just gold nanoparticles. (**B**) Gold nanoparticles and non-denatured glutathione complex. (**C**) Gold nanoparticles and non-denatured glutathione complex in the presence of 0.16% APTES. (**D**) Gold nanoparticles and denatured glutathione complex in the presence of 0.16% APTES.

In order to effectively describe the mechanisms by which the visual detection of denatured glutathione occurred, dynamic light scattering and FTIR-ATR measurements were performed on the samples. The resulting size and size distributions of just the gold nanoparticles, gold nanoparticles/GSH complex and denatured vs. non-denatured GSH in the presence of 0.16% APTES are shown in Fig. 4. Figure 4A shows the gold nanoparticles size and size distribution, for which the majority of the particles are about 18 nm. Upon the addition of the native GSH to the gold nanoparticles, the size and size distribution remains relatively constant with no appreciable change in comparison to the control.

This is supported by the absorbance spectra in Fig. 2A in which the AuNps/GSH sample max peak wavelength remained constant at approximately 500 nm; which is characteristic of just the gold nanoparticles alone. Upon the addition of 0.16% APTES to the solution we observed a distinct increase in the size and size distribution of the solution in Fig. 4D, after 2.5 hours of GSH heat stress. It is important to note that the size and size distribution increase is not due to particle growth, but aggregation of the gold nanoparticles.

**Figure 5:**
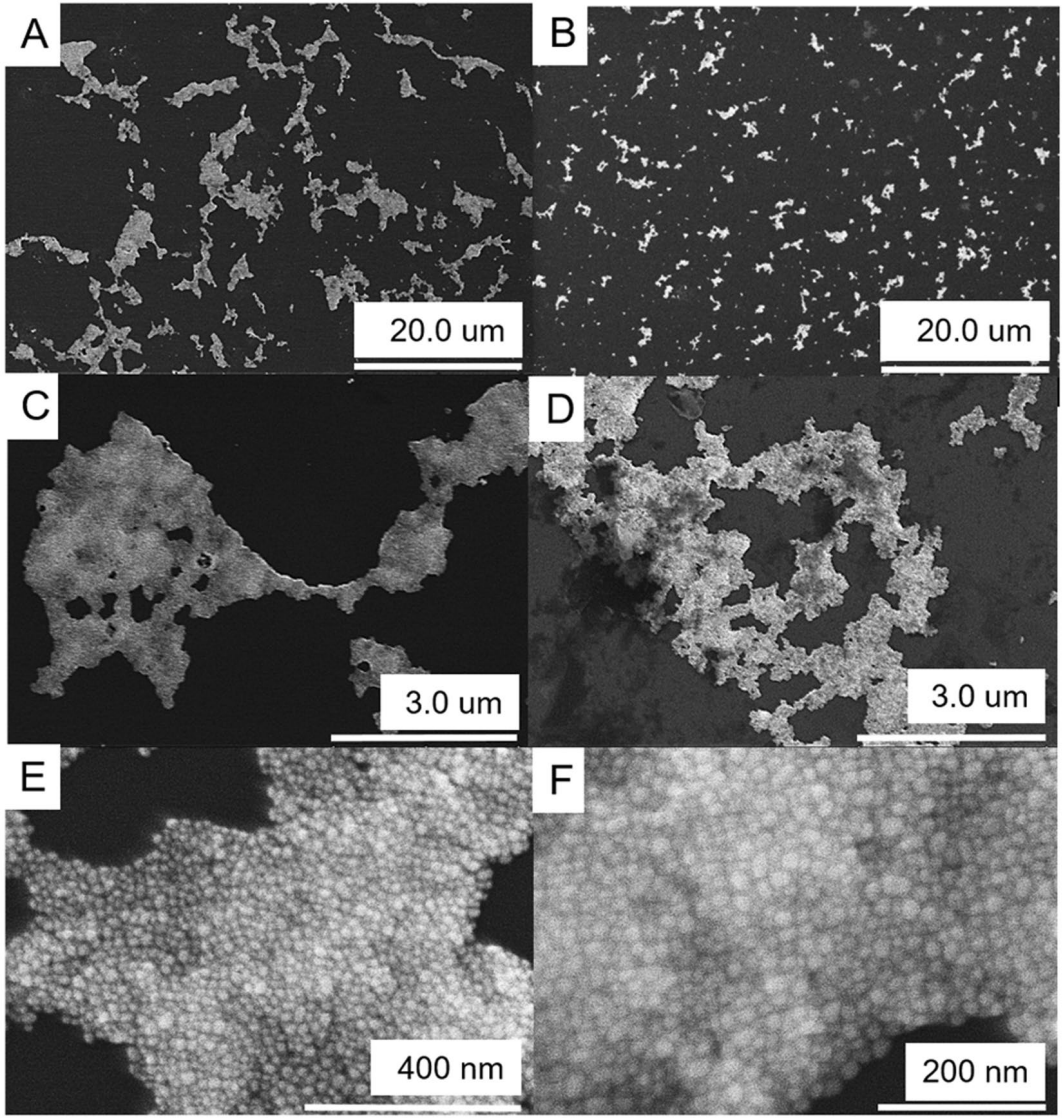
SEM images of the gold nanoparticles, glutathione and 0.16% APTES trials on a p-type silicon. The solutions were mixed, then deposited using a drop cast method and allowed to dry overnight. GSH 2.5 hour heat stress trial (**A**) 20.0 um, (**C**), 3.0 um, (**E**) 400 nm, (**F**) 200 nm. GSH 0 hour heat stress trial (**B**) 20.0 um and (**D**) 3.0 um. The samples were imaged using the HITACHI FE-SEM SU8010 instrument at 10.0 kV accelerating voltage and 5 milliamps.

The aggregation of the gold nanoparticles as a function of the concentration of denatured glutathione in the presence of APTES is further elucidated via the scanning electron microscopy (SEM) images shown in Fig. 5. In Fig. 5A we observe the presence of large scale aggregate formations, on average consisting of 10 um long aggregates for the denatured GSH trial. In contrast the non-denatured trial displayed in Fig. 5B shows much smaller aggregate masses, the majority of which are smaller than 5 um in size. In Fig. 5C we observed a densely packed mass for the denatured trial as opposed to a less densely packed sample within the non-denatured trials (Fig. 5D). Figure 5E and F are magnified images of the denatured GSH trial in which we observe uniformly sized gold nanoparticles and large scale AuNp clustering at 400 nm and 200 nm, respectively. The captured images in Fig. 5 further support the observed color change within the samples due to gold nanoparticles aggregation. The extent of this color change is as a function of the accelerated heat stress in the presence of APTES and can be attributed to aggregation and not individual particle growth.

**Figure 6:**
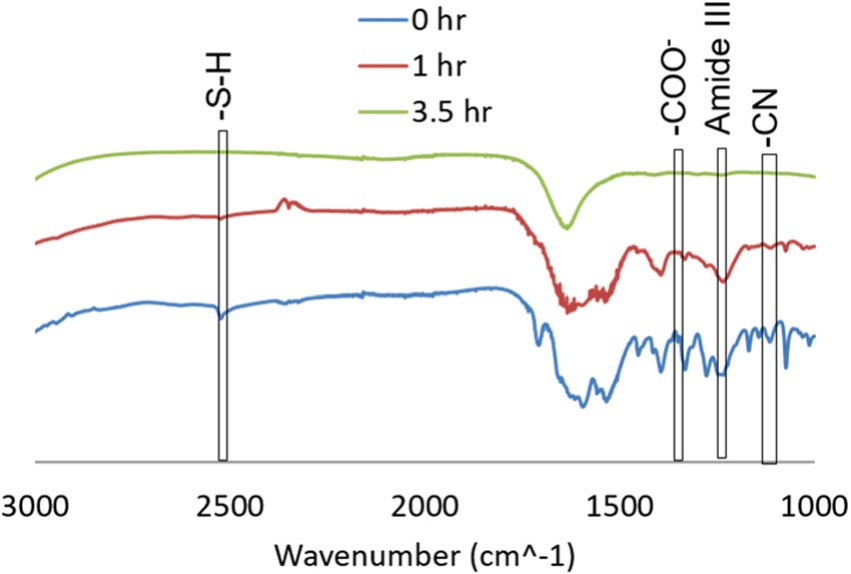
FTIR absorbance spectra of the glutathione peptide as a function of the accelerated heat stress exposure time.

Characterization of the glutathione during the accelerated heat stress was achieved through FTIR-ATR spectroscopy. The characteristic thiol functional group consisting of a hydrogenated sulfur, is present in the GSH sample that was not exposed to the accelerated heat stress. We also observe a COO- absorbance peak within the non-denatured GSH samples. As the GSH was heat stressed, 10 uL aliquots were removed and the absorbance spectra were monitored as a function of the exposure time. In Fig. 6, over the course of the accelerated heat stress study, the characteristic SH and COO- peaks decrease as a function of the applied heat stress. After 1 hour of degradation, the thiol and carboxylic acid groups decrease in intensity until the peaks are no longer distinguishable at 3.5 hours of heat stress.

**Figure 7:**
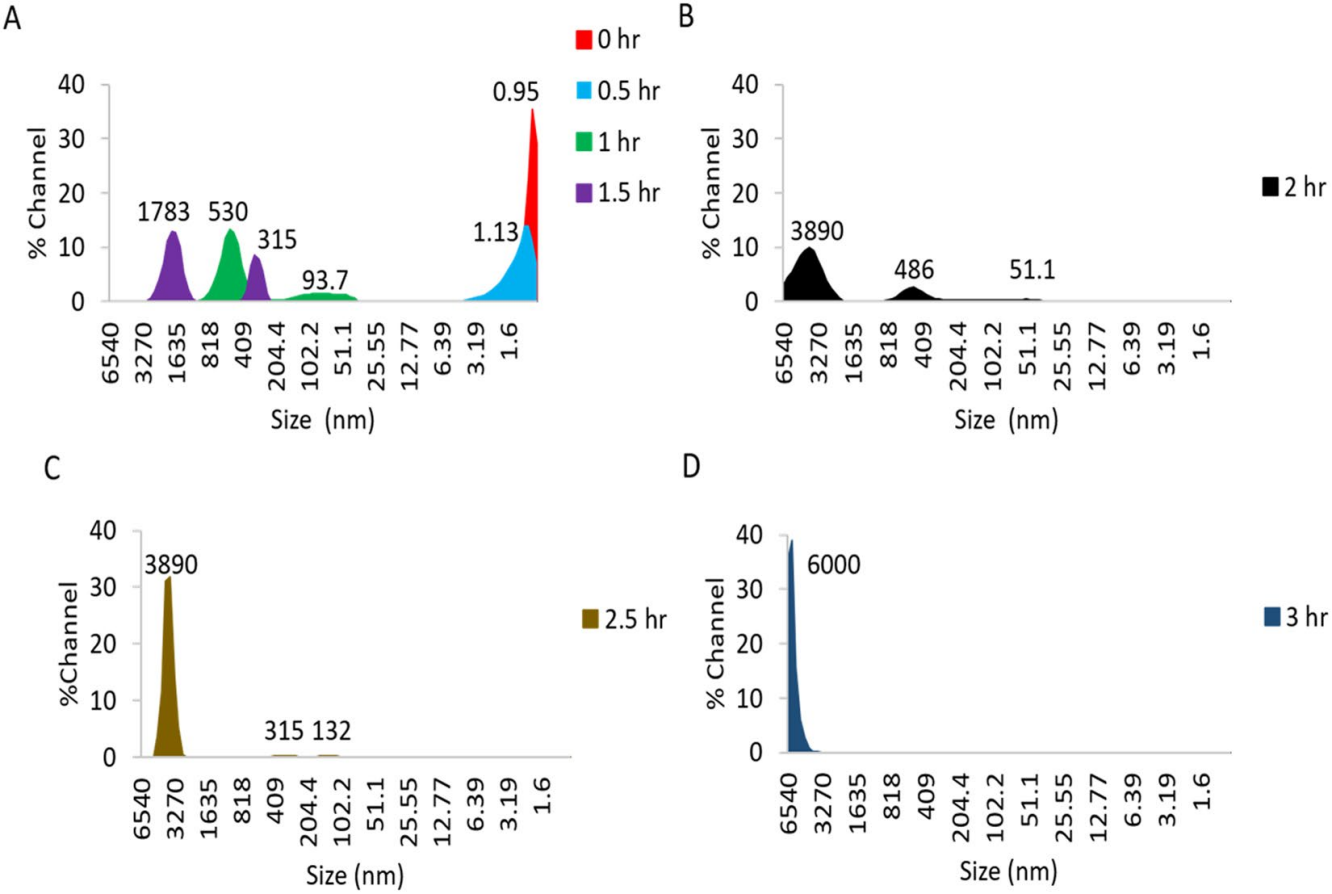
Size and size distribution plots of the glutathione trial samples over a 3 hour accelerated heat stress study at 70 °C, as measured by dynamic light scattering measurements.

Some of the major conventional techniques for measuring aggregation of biopharmaceuticals include; size exclusion chromatography and dynamic light scattering^41–45^. Size exclusion chromatography is very sensitive but often cannot process large scale aggregates or particulates^46–48^. Therefore, dynamic light scattering techniques are applied to determine size and size distribution of non-native biopharmaceuticals present within formulations. For the purpose of this study, a dynamic light scattering technique was used to characterize the protein aggregates. The authors would like to note that the actual size of glutathione in a non-aggregated state is outside of 0.87 nm detection limit for what can be measured accurately by the dynamic light scattering technique. However, in Fig. 7 we can still observe a systematic increase in the size of the GSH aggregates formed during 3 hours of accelerated heat stress. In Fig. 7A we observe in the 1 hour and 1.5 hour trials, the formation of large aggregates ranging in size from 93.7–530 nm and 315–1783 nm respectively. In Fig. 7B, the 2 hour heat stress trial displays two major peaks at 486 nm and 3890 nm. As the heat stress is continued to 2.5 hours, the peak at 486 nm disappears leaving a main peak at 3890 nm (Fig. 7C). The major peak in Fig. 7C increased in %channel and could be the result of the smaller aggregates at 486 nm (Fig. 7B) combining and contributing to the 3890 nm peak. This trend is continued and can be seen in Fig. 7D where the peak size begins to approach and push past the 6540 nm range limit of the instrument. Based on the dynamic light scattering results, the primary dependence of the detection mechanism is based on the extent of aggregation within the GSH samples formed during the accelerated heat stress study.

**Figure 8:**
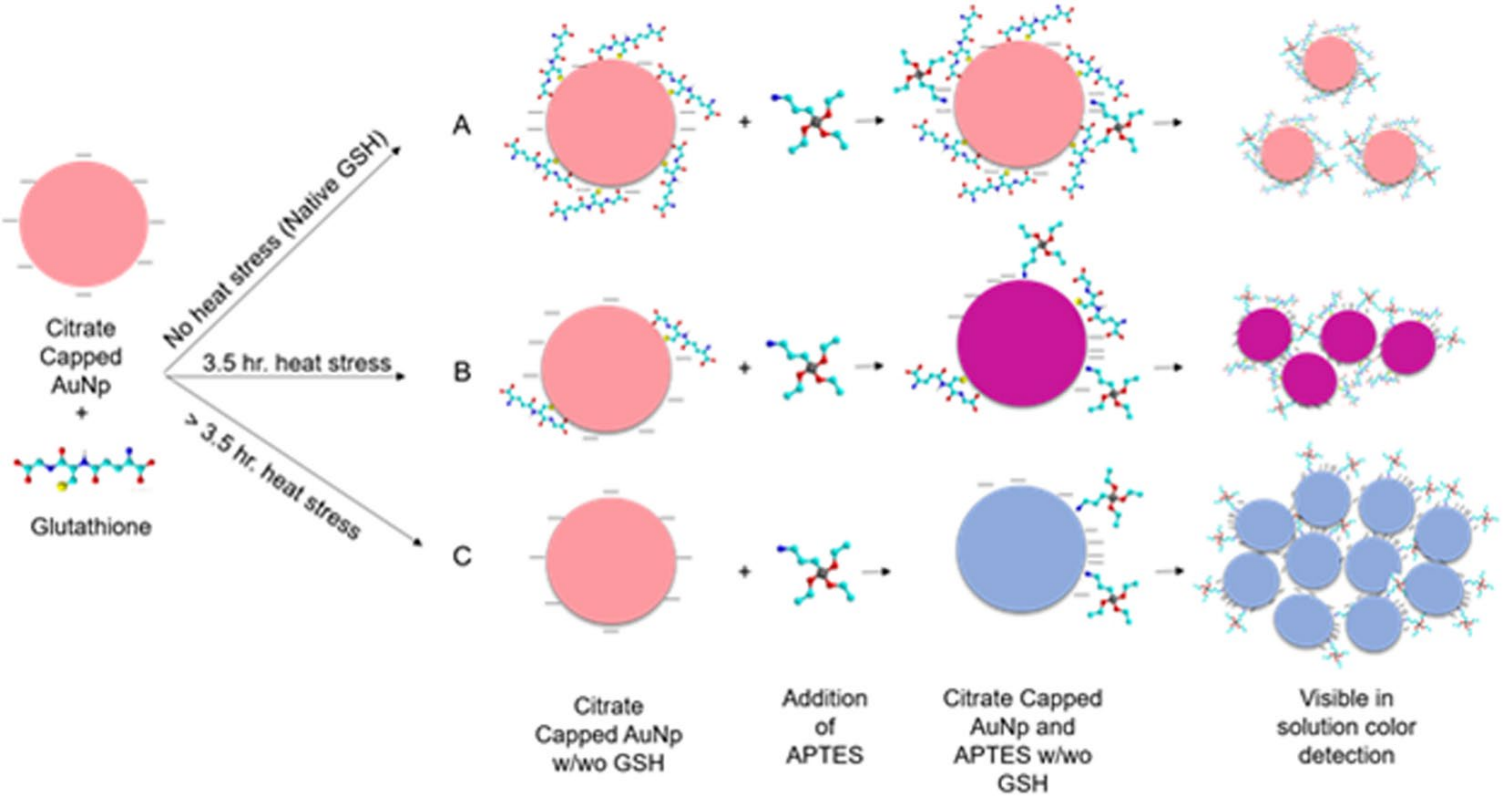
Proposed interactions of the citrate capped gold nanoparticles with denatured and non-denatured GSH in the presence of 0.1% APTES. The change in hue from the original red solution is modeled via gold nanoparticle clustering and aggregation as a function of the GSH accelerated heat stress.

Several papers have reported and discussed the intermolecular interactions of gold nanoparticles and glutathione. Depending on the pH of the solution, the SH or COO- functional groups will bind to the gold nanoparticles, producing an organically capped structure^49–52^. Combining this information with the phenomena of particle aggregation of the gold nanoparticles in the presence of APTES, the following mechanism for the observed visual detection is proposed. Figure 8 depicts the interactions of citrate capped gold nanoparticles with denatured and non-denatured solutions of GSH and 0.1% APTES. The gold nanoparticles synthesized utilizing gold chloride and trisodium citrate as a reducing agent, are stabilized via negatively charged citrate molecules through repulsive forces^53, 54^. The gold nanoparticle solution is approximately 5.6 in pH and so the GSH binds to the gold nanoparticles in two places via the SH functional group from the glutamate moiety and the COO- functional group^55, 56^. Changes in the hue from the original red solution of the AuNps is modeled due to clustering of the gold nanoparticles. Without the addition of APTES, the gold nanoparticle solution is relatively mono dispersed and maintains an optical absorbance of about 500 nm. These values when compared to literature were found to be comparable to other previously reported sizes and corresponding wavelengths^57–59^.

However, upon heat stressing the GSH, a decrease in the binding efficiency of the thiol and carboxylic acid functional groups are observed. Due to this decrease, strong dipoles are formed when APTES is added to the system. The gold nanoparticles then aggregate to mitigate the effects of these high energy dipoles. The APTES induced aggregation is effectively a function of the concentration of non-denatured GSH present in solution. In Fig. 8 we see that as the GSH is heat stressed, the visual changes from a red solution hue, to purple and then blue. This corresponds to the captured images in Fig. 3 and the UV-Vis absorbance spectra in Fig. 2. For the purpose of this work, only glutathione, a small antioxidant peptide was utilized. The authors would like to note that this work reflects only one type of biomolecule and other biomolecules may not yield the same effect. There may be several factors that affect the visual detection mechanism described above including; drug type and concentration, functional groups such as number of thiols present, protein structure (primary, secondary, etc.), size and overall stability.

To further explore the proposed visual detection system and determine assay range, extended experimentation must be performed for other drug types such as pegylated systems, monoclonal antibodies, and antibody conjugates. First, the allowable extent or concentration of denatured protein, peptide or antibody for a specific dosage must be identified analytically. Future work will also be centered on characterizing the presented system using analytical techniques such as high pressure liquid chromatography and ultra-high pressure liquid chromatography (size exclusion). Once a drug product’s range of allowable denaturation has been identified, the next step in this process requires full denaturation of the protein solution as confirmed via physical separations of aggregates or characterization of tertiary or secondary structural changes. Variations in the induced denaturation parameters can also be explored including, excessive shaking and a lower temperature profile over an extended time period. After complete denaturation, 100 uL of the biomolecule solution is added to 0.8 mL of the previously prepared gold nanoparticle solution. Subsequent absorbance studies are then performed to determine what concentration of APTES will induce a distinct color change. The APTES concentrations ranging from 0.1% to 10% will be tested using 100 uL of each respective concentration. A positive and negative control consisting of the non-denatured protein and AuNp solution exposed to APTES; coupled with a second solution of gold nanoparticles and APTES can be used to compare hues. The concentration of APTES that induces a distinct color change corresponding to the denatured protein solution will be specified for that particular protein and dose and can be used for future visual detection. Additional studies that incorporate parameters such as buffer type, surfactants, salt concentration, pH and other excipients should be evaluated for their impact on the proposed detection mechanism. The authors would like to note that the technique is not quantitative but may still be useful for aggregate evaluation.

## Discussion

Through this work we demonstrate the ability to visually detect denatured glutathione utilizing concentration dependent induced aggregation of gold nanoparticles. The need to visually detect denatured or non-native peptides is crucial as pharmaceutically industries begin to direct more efforts to drug discovery in the area of peptides. In addition, the presence of denatured biological molecules and macromolecules can cause a range of undesired biological responses. The presence of aggregates is a reoccurring concern in the pharmaceutical industries and should be addressed with both analytical and visual methods of detection. Therefore, this work proposes an initial proof of concept for the visual detection of heat stressed glutathione peptides. Continued proof of concepts with other biological analytes such as antibodies and antibody conjugates using this system is necessary.

## Methods

### Reagents

3-Aminopropyltreithoxysilane was procured from Sigma-Aldrich containing 98% purity. Gold chloride metallic salts with a 49% purity from Sigma-Aldrich was utilized for this study. A 99% ACS purity Trisodium citrate reagent used to reduce the gold chloride was obtained from Alfa Aesar. High purity grade reduced glutathione was purchased from Amresco. All chemicals were dissolved in deionized water (DI H2O) without employment of any further purification methods.

### Gold Nanoparticle Synthesis

The gold nanoparticles used in this study were prepared based on a simple one pot synthesis method^59^. Gold chloride and trisodium citrate were dissolved in separate vials containing deionized water. Explicitly 115 mg of trisodium citrate was dissolved in 10 ml of DI H_2_O and 40 mg of the gold chloride was contained in a separate vial. The gold (Au) precursor solution was heated to approximately 100 °C, after which the trisodium citrate was quickly injected into the reaction vial. After 10 minutes at a low boil, the gold nanoparticle solution was placed in an ice bath for 5 minutes under constant mixing. To increase the working sample volume, the gold nanoparticle solution was diluted 1:2 in deionized water.

### Peptide Preparation and Accelerated Heat Stress Study

A simple serial dilution was employed to obtain the 30.7 pg/mL GSH and 3.07 mg/mL GSH solutions in DI H20. Experimental trials were prepared by adding 100 uL of the 3.07 mg/mL GSH solution to 800 uL of the AuNps solution. After which, 100 uL of the respective APTES concentration is then added and subsequently characterized via ultraviolet visible spectroscopy. The 100 mL volume water bath consisted of a simple heating set up, in which a 500 mL glass beaker from VWR was filled and placed on a hot plate. The temperature of the system was monitored within the water bath. The 20 mL glass vial used to contain the peptide solution was also procured from VWR. To discourage evaporation of the water between sample aliquot removal, the vials were lightly capped as opposed to screwed on. In order to denature the peptides, 10 mL of the 3.07 mg/mL GSH solution was placed in a glass vial and exposed to an accelerated heat stressed environment via a hot water bath set to 70 °C. The peptide was allowed to denature over a 4 hour time period and 100 uL aliquots of the peptide solution was removed, mixed according to the above mentioned procedure and characterized. It is important to note that one minute before the peptide sample is taken out, the solution is mixed using a pipet to ensure well mixing. To properly capture the instantaneous color of the peptide, AuNp, and APTES solutions, a picture is taken directly after the addition of APTES and then loaded onto the UV-Vis.

### Characterization

To pinpoint the morphological changes within the sample as a function of the extent of GSH denaturation, scanning electron microscopy (SEM) were taken using the HITACHI FE-SEM SU8010 instrument at 10.0 kV accelerating voltage and 5 milliamps. As the detection system is based on visual changes in the solution hues, the LAMBDA 950 UV/Vis/NIR Spectrophotometer by PerkinElmer was employed in absorbance mode to quantify the max peak wavelength shifts. The parameters used for UV-Vis spectra consisted of a 2 nm slit width and scan speed of 480.23 nm/min over a 400 nm range starting at 800 nm. To formally quantify morphological changes in the system due to aggregation of the gold nanoparticles, a dynamic light scattering technique was used to elucidate the size and size distribution of the gold nanoparticle, GSH and APTES systems via the Nanotrac Wave by Microtrac. The 60 second trials runs on the Nanotrac Wave by Microtac used a 780 nm laser diode. FTIR spectroscopy was employed to determine denaturation of GSH via a transmission spectrum over the range of 3000–1000 (cm^−1^), using a 0.5 (cm^−1) resolution. This was achieved using the PerkinElmer Spectrum Two Spectrophotometer and Spectrum software application version 10.03.06.0100.
